# Supplementary material for: Publication games: In the web of reciprocity
Source: PLoS One. 2022 Oct 26;17(10):e0270618. doi: 10.1371/journal.pone.0270618 (PMC9604877; doi:10.1371/journal.pone.0270618)
Supplement: Attachment — Submitted filename: PP-MS_metrics-plosone-rebutal.docx [file pone.0270618.s001.docx]

Alberto Baccini, Ph.D.
Academic Editor
PLOS ONE

**RE: PONE-D-22-16973, “Publication games: in the web of reciprocity”**

Dear Dr. Baccini,

Many thanks for effort to deal with my manuscript and your overall positive opinion. Below I respond to all of your and the reviewer’s comments (typed in italic) in details. Line numbers corresponds to the marked-up copy of the MS.

*1) The paper omitted completely the technicalities of simulations. I think that you should add information about the technique and code you used. This probably led to some difficulties in reading Figure 5 and Figure 6.*

Thank you for pointing out this deficiency in the MS. Now I added a paragraph (l. 171-179) about the simulations. During this I recognised that simulations were performed only once so I repeated them 1000 times. As a result my conclusion on Fig 6 changed a bit (l. 194-202), so I performed more simulations to clarify this which resulted in a new figure (Fig 7) and some more text (l. 203-231). These results show that the application of 1/n rule can have different effect of authors of different prior productivity. I now discuss these findings in the Conclusions (l. 263-286).

*2) In all the figures, the size of node changes. I assume it is proportional to the weighted number of papers authored by a node author. Moreover, the use of different colours in the lower panels of figures 5- and 6 is not explicitly addressed. Finally, the choice of representing a specific cartel in the graph is not commented at all.*

You are right again here. I added to the figure legends that node size is proportional to the number of coauthors (degree). Use of different colours are also clarified in the legends of Fig 5 and 6 as well as the use of specific cartels are justified here.

*3) There are some minor inconsistencies in the notation (use of ’ instead of ’).*

These are corrected now.

*4) As for discussion and policy indication, in I understand correctly your analysis, the suggestion that the adoption of the rule of 1/n may lead to self-purification is not consistent with your results.. The conflict of interest induced by the rule is valid only for authors of different productivity. As you explicitly stated, if a group of similar productivity authors forms a cartel, it boosts the productivity of its members. Hence, there is a clear strategy that similar-productivity authors may adopt for gaining positions in the publication game. Moreover, in a dynamic game, where people of similar productivity gain position, also the higher-productivity authors may have an interest to form cartels in view of avoiding being reached by cartels formed by lower-productivity authors.*

You are absolutely right here, I modified the Conclusions accordingly taking into account the new results presented in Fig 7 as well.

*Reviewer #1: The paper deals with an important distortion in the production of science, which is the formation of cartel publications. Besides individual incentives associated with higher productivity leading to higher salaries and promotion, there are also institutional incentives to push faculty to increase their research output, such as public founding agencies and/or college associations membership. See, for example, Besancenot et al. (2009) Why Business Schools do so much research: A signaling Explanation (2009) Research Policy 38, 1093-1101 Faria, J. and F. Mixon (2022) Opportunism vs. excellence in academia: Quality accreditation of collegiate business schools (2022) American Business Review, open source.*

Thank you for pointing out these wider implications. Faria & Mixon is referenced now to call attention for this phenomenon too.

I hope that after these modifications the MS is now suitable for publication in PLOS ONE.

Sincerely yours,
Zoltan Barta
